# Mohs Surgery for Periocular Basal Cell Carcinoma Without a Mohs Surgeon: The First Series in Hong Kong

**DOI:** 10.7759/cureus.36235

**Published:** 2023-03-16

**Authors:** Regine Chan, Chi-lai Li, David Liu, Nai Ming Luk, Alvin Young, Paul Choi, Kelvin Chong

**Affiliations:** 1 Department of Ophthalmology and Visual Sciences, Prince of Wales Hospital, Sha Tin, HKG; 2 Department of Ophthalmology and Visual Sciences, The Chinese University of Hong Kong, Central Ave, HKG; 3 Dermatology Service, Prince of Wales Hospital, Sha Tin, HKG; 4 Department of Anatomical and Cellular Pathology, Prince of Wales Hospital, Sha Tin, HKG; 5 Department of Ophthalmology, The Chinese University of Hong Kong, Central Ave, HKG

**Keywords:** asians, eyelid, periocular, basal cell carcinoma, mohs surgery

## Abstract

Purpose

To report the first series of Mohs micrographic surgery (MMS) in Hong Kong, where the roles of a Mohs surgeon were shared and coordinated by a “mobile” surgeon.

Methods

Design: Prospective non-comparative interventional case series.

Subjects: 20 consecutive Chinese patients (10 male, age 78.5+10.4 years, range 55-91 years) with primary periocular basal cell carcinoma (pBCC) referred to the university oculoplastic unit between October 2007 and August 2013.

Intervention: MMS were conducted according to a streamlined standard operating procedure emphasizing surgeon-driven mapping, specimen orientation, and on-site clinico-histological correlation with the dermatopathologist at the frozen-section laboratory.

Main Outcome Measures: Clinical and histological characteristics of tumors, layers of Mohs procedures, complications, and biopsy-confirmed recurrence at the same location.

Results

All 20 patients received MMS as planned. Sixteen pBCCs (80%) were diffusely pigmented, and three (15%) were focally pigmented. Sixteen were also nodular. The average tumor diameter was 7+3 (3-15) mm. Seven (35%) were within 2 mm of the punctum. Histologically, 11 (55%) were nodules, and four (20%) were superficial. An average of 1.8+0.8 Mohs levels were performed. Apart from the initial two patients, who required four and three levels, respectively, seven (35%) patients were cleared after the first level of MMS using a 1mm clinical margin. The remaining 11 patients required two levels with an additional 1-2mm margin, but only focally as guided histologically. Defects in 16 patients (80%) were reconstructed by local flaps, two by direct closure, and two with pentagon closure. Among the seven patients with pericanalicular BCC, three patients had their remaining canaliculi successfully intubated, while two developed stenotic upper and two lower punctae postoperatively. One patient had prolonged wound healing. Three patients had lid margin notching, two had medial ectropion, one had medial canthal rounding, and two had lateral canthal dystopia. No recurrence was detected at a mean follow-up of 80+23 months (43 to 113 months) in all patients.

Conclusions

MMS was successfully introduced in Hong Kong without a Mohs surgeon. Providing complete microscopic margin control and preserving tissues, it was proven to be a valuable treatment option for pBCC. Our multidisciplinary protocol demonstrated that these merits are possible and warrant validation in other resource-limited healthcare settings.

## Introduction

Basal cell carcinoma (BCC) is the most common malignant neoplasm in humans [1]. While it is more prevalent in fair-skinned Caucasians, Asians are not spared with its growing incidence worldwide [2-3]. Among the Chinese in Hong Kong, the local Cancer Registry recorded a 90% increase in the annual incidence of non-melanoma skin cancers (NMSC) from 1990 to 2010, of which 75% were BCC in 2007 [4]. In Taiwan, there was a steeply increasing incidence of eyelid cancers from 1979 to 1999, with BCC dominating the trend [5]. 10% to 15% of all BCC occur in the periocular region, part of the “H” zone along the embryonic fusion planes on the face, where it is considered high risk for incomplete treatment and recurrence [6-7]. BBC is treated by surgical excision, destructive modalities (e.g., curettage, cryosurgery), or non-surgical options (e.g., topical or radiation therapy) [8]. Since BCC often shows irregular, asymmetric subclinical growth, unmonitored excision using an arbitrary surgical margin (i.e., wide local excision) or standard “bread-loafing”, frozen-section margin control poses the risk of incomplete excision and recurrence [9]. Recurrent BCCs are larger, have higher recurrence rates, have more aggressive histology, and have more extensive subclinical infiltration [10].

Mohs micrographic surgery (MMS) combines microscopic (micro-) monitoring of the entire excised margin around the lesion with pictorial mapping (-graphic) [11]. It is considered by many to be the treatment of choice for both primary and recurrent pBCCs [12]. However, it has not been available in Hong Kong. Local standard-of-care includes unmonitored resection with variable (3-4mm) margins as wide local excision; vertical, “bread-loafing” frozen-section monitored resection; or radiotherapy for patients unsuitable for surgeries [13]. We reported our 10-year experience in setting up the first MMS in Hong Kong through a multidisciplinary approach to fulfill the different roles of a Mohs surgeon, coordinated by a “mobile” surgeon.

## Materials and methods

This is a prospective non-comparative interventional study of 20 consecutive patients with biopsy-proven peri-ocular BCC who underwent MMS in a university oculoplastic unit in Hong Kong between October 2007 and August 2013. The study followed the Declaration of Helsinki, and all subjects provided informed consent, which was approved by the research ethics committee of the Chinese University of Hong Kong.

Inclusion and exclusion criteria

Periocular BCC was defined as those involving the medial or lateral canthus, upper or lower eyelid, or a combination of these sites (complex), with or without eyelid margin involvement. Every patient with suspicious growth underwent an incisional biopsy. Biopsy-proven periocular BCC was offered a fast-track referral within two weeks. Treatment options were explained, and those who opted for MMS were recruited. Patients with residual or recurrent BCC, those who refused MMS, or those with incomplete follow-up were excluded.

Clinical assessment

Symptoms and their duration, medical and drug histories, family or personal history of (skin) cancer, and any predisposing condition were documented. Location, distance from anatomical landmarks (lid margin, punctum, canthus), size (maximal dimension in mm), presence of pigmentations, morphological subtypes (nodular, nodulo-ulcerative, morphoeaform), and Fitzpatrick skin type were recorded.

Standard operating procedure for fresh-tissue Mohs micrographic surgery (MMS)

MMS was carried out after local infiltration with 1% xylocaine and adrenaline (1:200000) mixed with 0.5% Marcaine (1:1) and hyalase. 1 mm of clinically normal skin was incised around the tumor border, followed by undersurface excision at the desired depth, typically at the orbicularis muscle plane with a #11 blade. Reference marks were made with sutures of different colors that were left long and untied. Hemostasis was secured, and the wound was dressed while the patient was transferred to a waiting area. A pictorial tissue map was constructed and presented to the dermatopathologist at the frozen-section laboratory by the “mobile” surgeon. After confirming the orientation, the specimen was inked and positioned with the undersurface and the peripheral margin lying flat on a glass slide. The cutting chuk was layered with optimal cutting temperature (OCT) material and snap-frozen. The specimen-chuk complex was detached from the glass slide and positioned with the undersurface and the flattened peripheral margin of the tumor facing the microtome blade. Frozen sections were generated containing the circumferential peripheral (epidermal) and deep margins at the first few cuts for hematoxylin and eosin staining. Additional cuts were made until all epidermal margins were adequately assessed. Slides were reviewed by the dermatopathologist and the “mobile” surgeon together. The “mobile” surgeon’s personal attendance ensures the specimens are precisely mapped at the frozen-section laboratory, and microscopic findings are correlated surgically onto the patient by a Mohs surgeon. Subsequent layers were removed at 1-2mm per level depending on tissue status, but only where the residual tumor was visible. The Mohs defects were reconstructed by direct closure, flaps, and grafts, as indicated.

## Results

Twenty ethnic Chinese patients (10 females) were operated on. The mean overall age at diagnosis was 78.5+10.4 years (range: 55-91): 76 for males and 81 for females. One patient had a documented history of BCC of the fellow eyelid. Another patient was on long-term azathioprine for pemphigoid. None had Gorlin syndrome. All patients were Fitzpatrick Skin Type III or IV. The tumor was present for a mean of three years (1-10 years) with an average diameter of 7.3 mm (2.5-15 mm). Clinically, 16 tumors were nodular, and four were ulcerative/nodulo-ulcerative. Sixteen (80%) were diffusely, and three were focally pigmented. The only non-pigmented BCC showed focal pigmentary deposition and incontinence microscopically. 11 were on the right. Eleven tumors were from the lower eyelid, five from the upper eyelid, and four at the medial canthus. Seven are affected or located within 2 mm of the punctum (peripunctal/pericanalicular).

**Figure 1:**
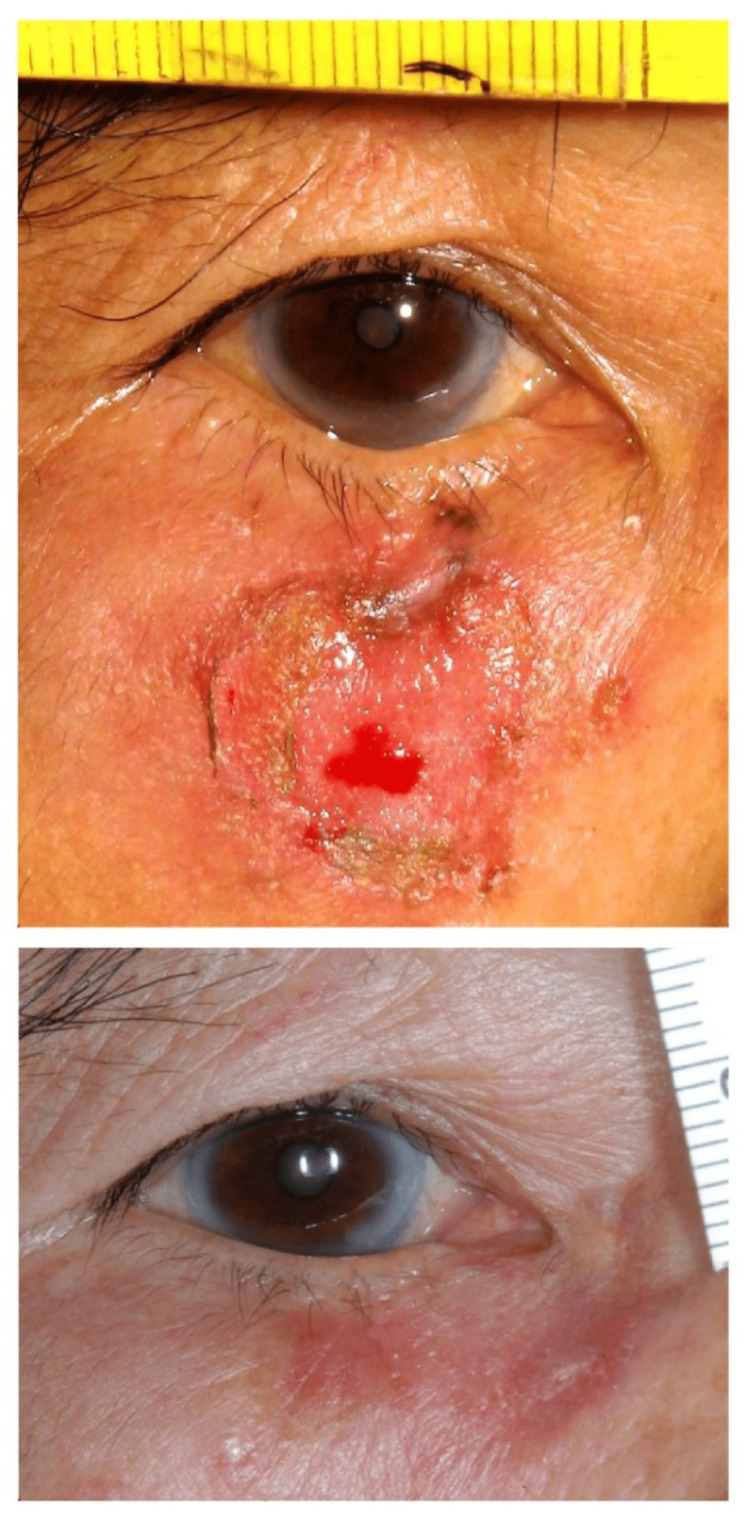
Preoperative and three-month postoperative photos of our first patient with a right lower lid pigmented BCC complicated by a surrounding herbal medication-related skin ulcer, four levels of MMS done. BCC: Basal cell carcinoma, MMS: Mohs micrographic surgery

An average of 1.8+0.8 Mohs layers were performed. Apart from the initial two cases requiring four and three levels, respectively, seven (35%) patients were cleared after the first Mohs level using a 1mm clinical margin. The remaining 11 patients required two levels with an additional 1-2mm of margin removed, but only focally. The first patient presented with a large skin ulcer surrounding the tumor after the self-application of herbal medicine and was cleared after the fourth layer (Figure 1) [14].

**Figure 2:**
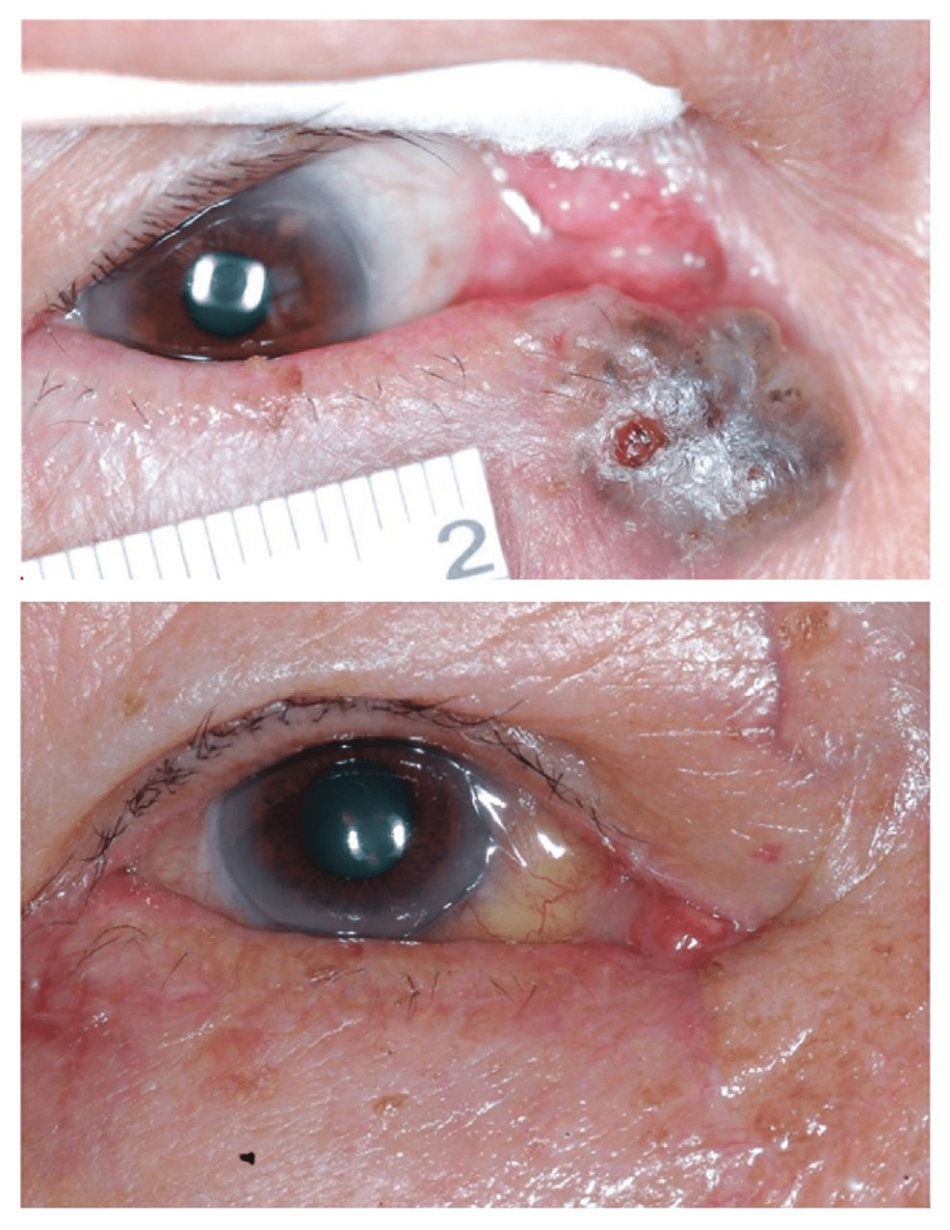
Preoperative and two-month postoperative photos of our second patient. Three levels of MMS procedures done with failed intraoperative canalicular intubation. MMS: Mohs micrographic surgery

The second patient with a large peripunctal/pericanalicular pigmented BCC had deep canalicular involvement and was cleared with three levels (Figure 2).

Histologically, 11 (65%) were nodules, four were superficial, and two were diffuse. The remaining three had mixed features: two superficial with focal areas of diffuse pattern and one nodular with a focal area of diffuse subtype. None showed peri-neural invasion.

**Figure 3:**
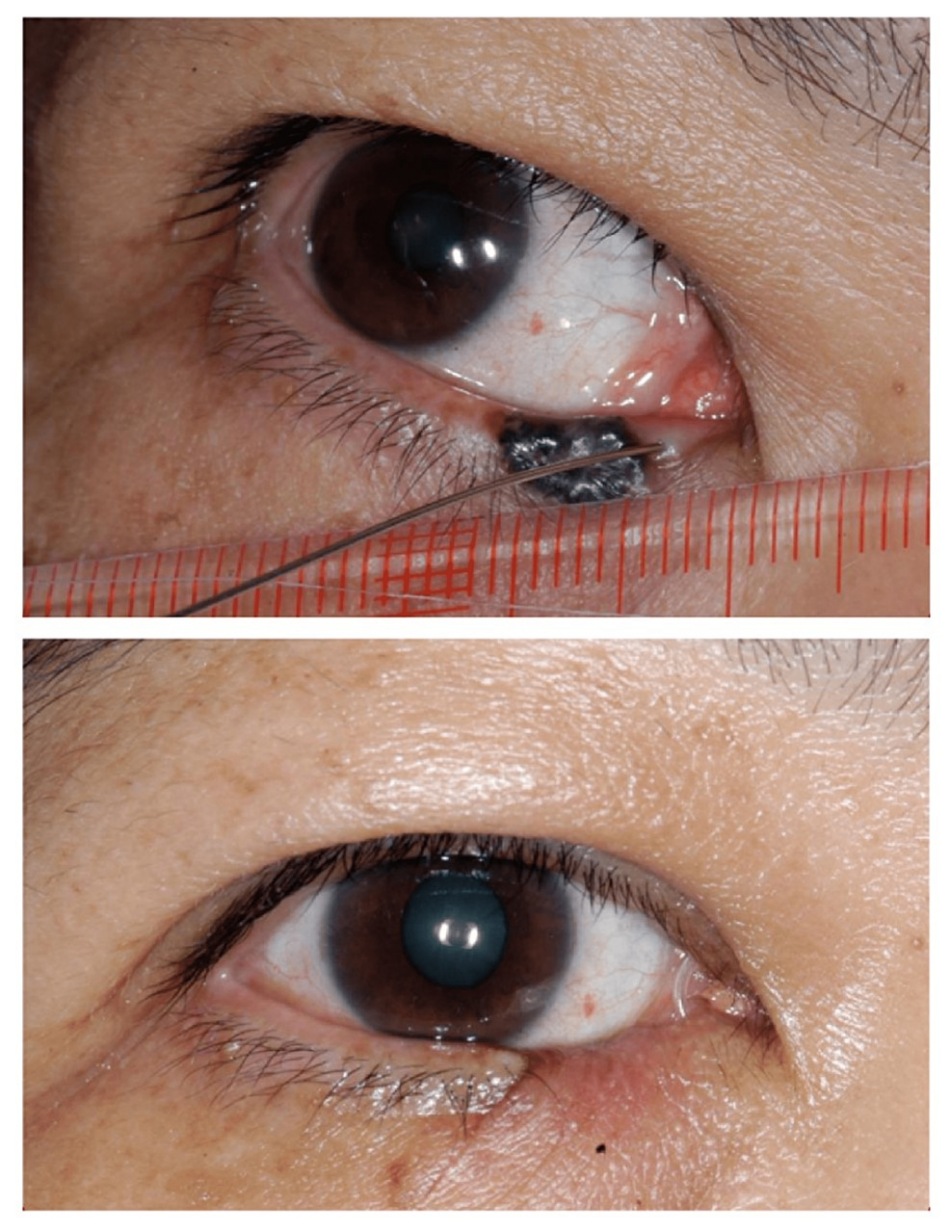
Preoperative and two-month postoperative photos of a patient with right lower lid peripunctal pigmented BCC and successful intraoperative canalicular intubation. BCC: Basal cell carcinoma

Sixteen (80%) defects were reconstructed by local flaps, two by direct closure, and two with pentagon closure. One patient required an additional free tarsal graft for extensive posterior lamellar involvement. Among the seven patients with peripunctal BCC, three patients were successfully intubated intraoperatively. Bicanalicular stents were removed at eight weeks postoperatively (Figure 3).

**Figure 4:**
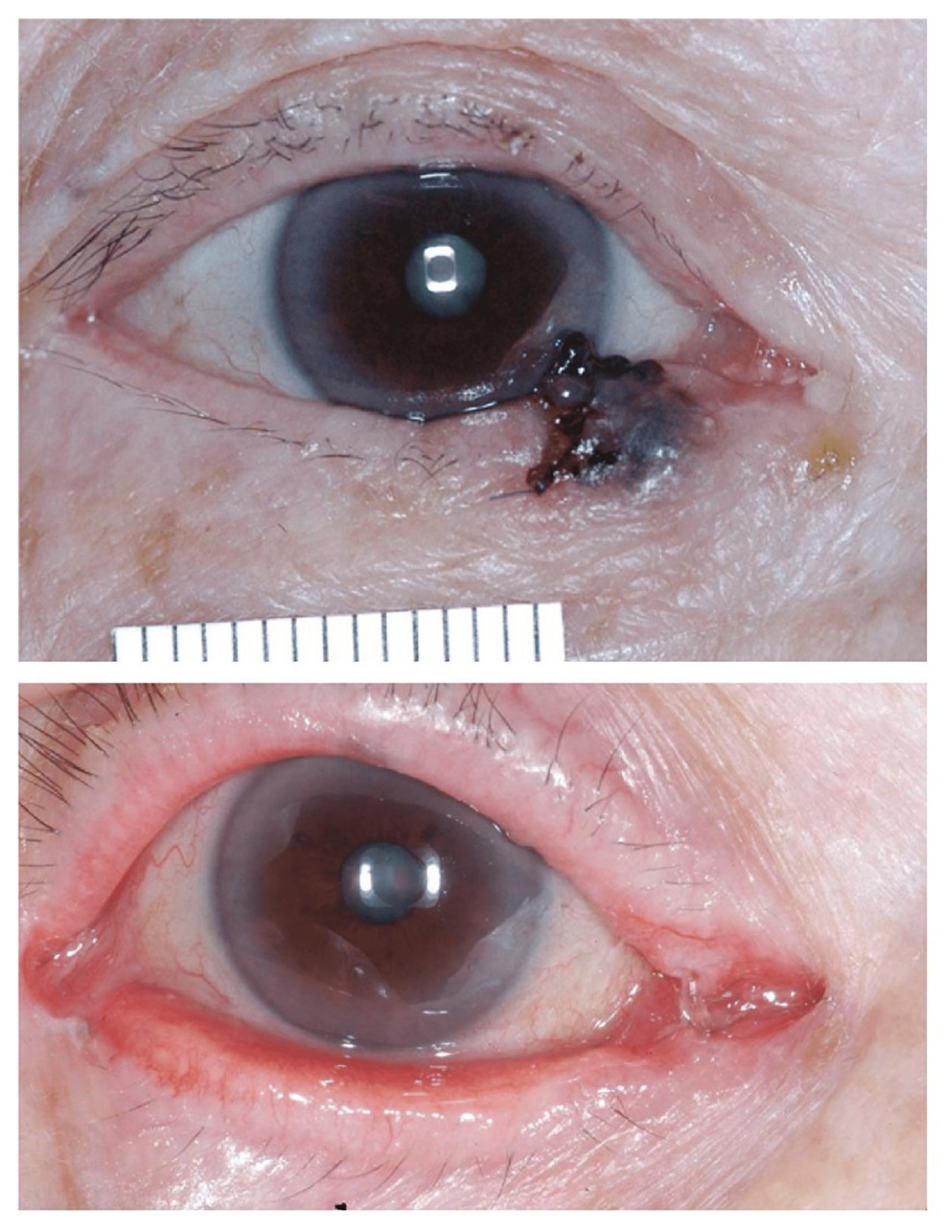
Preoperative and two-month postoperative photos of a patient with pigmented peripunctal BCC and silicone intubation in-situ. Note the mild lid margin notching. BCC: Basal cell carcinoma

**Figure 5:**
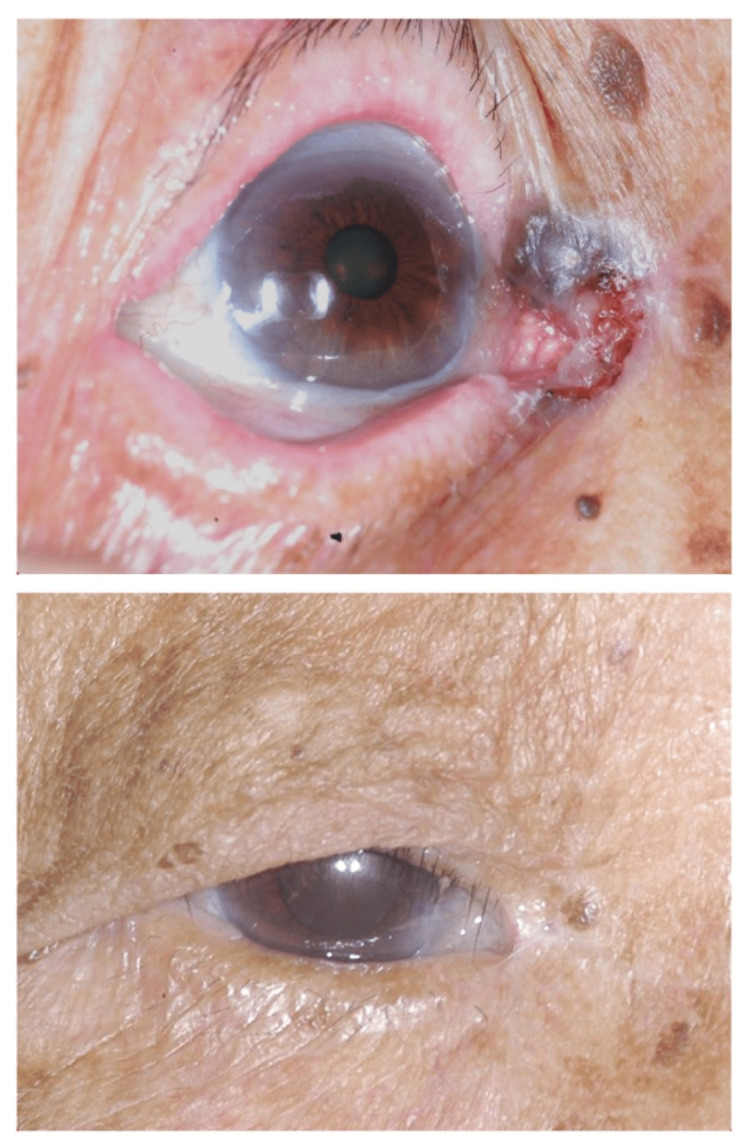
Preoperative and three-month postoperative photos of the right lower lid peripunctal/pericanalicular pigmented BCC, showing postoperative medial ectropion. The lacrimal system was patent after silicone tube removal BCC: Basal cell carcinoma

**Figure 6:**
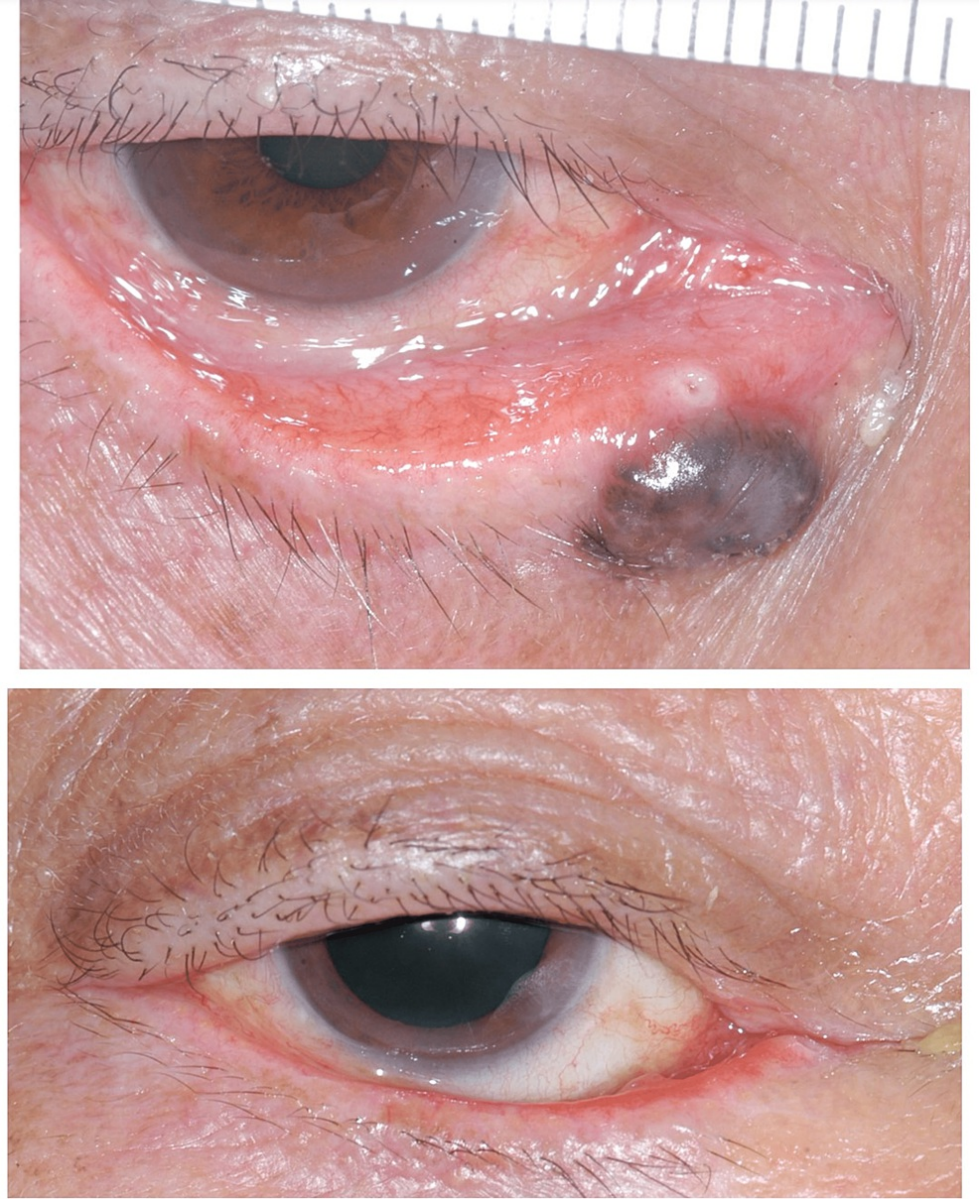
Preoperative and three-months postoperative photos of a patient with right medial canthal, upper and lower peripunctal and pericanalicular pigmented BCC with showing postoperative medial canthal rounding and punctal stenoses. Both canaliculae were removed at the second Mohs level. BCC: Basal cell carcinoma

The remaining four patients developed punctal stenosis (two uppers and two lower) postoperatively. Our first patient had prolonged healing related to a pre-existing herbal medication-related skin ulcer (Figure 1). Three patients had mild lid notching (Figure 4), two had medial ectropion (Figure 5), one had medial canthal rounding (Figure 6), and two had lateral canthal dystopia.

All patients refused further intervention as they were not symptomatic. No recurrence was detected for all patients at a mean follow-up of 80+23 months (43 to 113 months).

## Discussion

Patients with pBCC are at higher risk for incomplete excision, subsequent recurrence, and significant postoperative functional and aesthetic morbidities [6-12]. Although the recent Cochrane review did not conclude whether wide-local excision or MMS resulted in a lower recurrence or complication rate for pBCC, the latter still reported the lowest recurrence rate to date amongst all treatment modalities for pBCC [8-12]. These are related to the 100% circumferential and deep margin sampling by specimen flattening and horizontal sectioning in MMS, compared to less than 1% of margin sampling during standard “bread-loafing” vertical step sectioning. The reliability of MMS was attested by the five-year cure rates of 99% in 1,773 cases of BCC by Dr. Mohs [11] and validated by a 0% 5-year recurrence rate of primary periocular BCC in Australia’s national database [15]. It was proven to be tissue-sparing even in small, nodular BCC in a randomized controlled trial [16]. The randomized controlled trial of 405 high-risk facial BCCs conducted in the Netherlands also confirmed fewer cases of recurrence with MMS compared to treatment with wide local excision [17]. The 10-year recurrence rates were 4% after MMS and 12.2% after surgical excision (p=0.1) [17].

Modifications of histological preparation in MMS or frozen section techniques [18-20] have been reported, including a multidisciplinary team approach involving the Mohs (ablative) and the reconstructive surgeon in the periocular region [21]. While we concur with the rationale and use of MMS for the treatment of pBCC, we have been working out how it should be adopted in Hong Kong, where non-melanoma skin cancer is of lower incidence and establishing a dedicated Mohs service is not cost-effective [22]. Here we propose a surgeon-driven, multidisciplinary protocol based on available frozen-section pathology services. To ensure cost-effectiveness, only patients with biopsy-proven pBCC were referred through a fast-track system, and the operating room could be used by other surgeons during specimen processing. While the Mohs surgeon excises, maps, and reviews the specimen personally, thus minimizing any transcription error, the “mobile” surgeon personally attends to the critical steps, including specimen mapping, orientation, and handling. To ensure accurate clinicopathological correlation on marginal clearance, slides were immediately reviewed with the dermatopathologist for the surgical decision. With all pBCCs in this series smaller than the diameter of a regular-sized cryostat chuck (approximately 30mm in diameter), the single section method was adopted to avoid specimen division [23]. Our protocol achieved a shorter turn-around time than standard frozen-section in our hospital, which was further expedited by the “mobile” surgeon delivering the specimens personally. Slides were available within 20 minutes after tissue removal. Once all margins were examined, the remaining specimens were subject to standard paraffin sectioning for accurate histological subtyping.

BCC is more often pigmented in the skin of people of color. In Hong Kong, it was estimated that 60% of all BCCs were clinically pigmented [24]. In a large US series, Sexton et al. found 7% of their BCC pigmented. They further showed that pigmented BCC were more likely to be completely excised than the non-pigmented ones adjusted for histological subtypes and that there was a negative association between infiltrative (aggressive) histologies and pigmentation [25]. Aoyagi and Nouri from Japan found a significantly smaller mean surgical margin by MMS in pigmented than non-pigmented BCC (3.32 versus 5.33 mm) for tumors smaller than 2 cm in diameter with non-aggressive histological subtypes (i.e., nodular or superficial) [26]. In this Chinese series, 95% of our pBCC were pigmented, and 90% of our patients required no more than 2 Mohs levels, i.e., up to only a focal 3-mm surgical margin, while seven tumors were cleared by a 1-mm circumferential margin. However, there was inadequate evidence to extrapolate our findings to the use of a smaller surgical resection margin for pigmented pBCC by unmonitored or standard bread-loaf frozen-section monitored excision.

Seven out of the 20 (35%) pBCC were within 2mm of the punctum or along the pericanalicular area, forming the largest series in the literature [27]. Located in the medial canthus, they carry not only a higher risk of recurrence and orbital infiltration but also canalicular involvement [28]. MMS offered both oncological clearance and the best chance of preserving the proximal lacrimal system, which would otherwise be removed during a wide local excision.

The results of this study should be further validated involving different oculoplastic units and a larger patient cohort for longer follow-up. We also propose the use of digital and mobile technologies to facilitate the “mobile” surgeon as reported during standard MMS [29].

## Conclusions

Although most of the pBCCs in this series were pigmented, nodular, and of less aggressive histological subtypes, one-third were pericanalicular or peripunctal and posed reconstructive challenges. The successful adoption of MMS ensures not only complete margin control but also superior tissue preservation. With careful planning, the roles of a Mohs surgeon can be satisfactorily shared, coordinated, and motivated by a “mobile” surgeon, benefiting many resource-limited healthcare systems.
